# Adult-onset Alexander disease with typical "tadpole" brainstem atrophy and unusual bilateral basal ganglia involvement: a case report and review of the literature

**DOI:** 10.1186/1471-2377-10-21

**Published:** 2010-04-01

**Authors:** Michito Namekawa, Yoshihisa Takiyama, Junko Honda, Haruo Shimazaki, Kumi Sakoe, Imaharu Nakano

**Affiliations:** 1 Department of Neurology, Jichi Medical University, Tochigi, Japan; 2 Department of Neurology, Interdisciplinary Graduate School of Medicine and Engineering University of Yamanashi, Yamanashi, Japan; 3 Department of Hematology, Interdisciplinary Graduate School of Medicine and Engineering University of Yamanashi, Yamanashi, Japan

## Abstract

**Background:**

Alexander disease (ALX) is a rare neurological disorder characterized by white matter degeneration and cytoplasmic inclusions in astrocytes called Rosenthal fibers, labeled by antibodies against glial fibrillary acidic protein (GFAP). Three subtypes are distinguished according to age at onset: infantile (under age 2), juvenile (age 2 to 12) and adult (over age 12). Following the identification of heterozygous mutations in *GFAP *that cause this disease, cases of adult-onset ALX have been increasingly reported.

**Case Presentation:**

We present a 60-year-old Japanese man with an unremarkable past and no family history of ALX. After head trauma in a traffic accident at the age of 46, his character changed, and dementia and dysarthria developed, but he remained independent. Spastic paresis and dysphagia were observed at age 57 and 59, respectively, and worsened progressively. Neurological examination at the age of 60 revealed dementia, pseudobulbar palsy, left-side predominant spastic tetraparesis, axial rigidity, bradykinesia and gaze-evoked nystagmus. Brain MRI showed tadpole-like atrophy of the brainstem, caused by marked atrophy of the medulla oblongata, cervical spinal cord and midbrain tegmentum, with an intact pontine base. Analysis of the *GFAP *gene revealed a heterozygous missense mutation, c.827G>T, p.R276L, which was already shown to be pathogenic in a case of pathologically proven hereditary adult-onset ALX.

**Conclusion:**

The typical tadpole-like appearance of the brainstem is strongly suggestive of adult-onset ALX, and should lead to a genetic investigation of the *GFAP *gene. The unusual feature of this patient is the symmetrical involvement of the basal ganglia, which is rarely observed in the adult form of the disease. More patients must be examined to confirm, clinically and neuroradiologically, extrapyramidal involvement of the basal ganglia in adult-onset ALX.

## Background

Alexander disease (ALX) (OMIM #203450), originally described by Alexander in 1949 [1], is a rare and fatal disease of the central nervous system caused by astrocyte dysfunction [2,3]. The pathological hallmark of the disease is the accumulation of ubiquitinated intracytoplasmic inclusions in astrocytes, called Rosenthal fibers, which are composed of glial fibrillary acidic protein (GFAP), the main intermediate filament of astrocytes, in association with the small heat shock proteins, HSP27 and αB-crystallin [4].

The clinical features of typical infantile-onset ALX, with onset before the age of two, include megalencephaly, seizures, spastic paresis and psychomotor deterioration with leukoencephalopathy characterized by white matter abnormalities predominating in the frontal lobes. As cases accumulate, however, atypical patients have also been described. Adult-onset ALX, with onset over the age of 12, is characterized by more slowly progressive bulbar or pseudobulbar palsy, spastic paresis, ataxia, palatal myoclonus and essentially normal psychic and intellectual functions. Juvenile-onset ALX, with onset between age 2 and 12, bridges the gap between infantile and adult forms of the disease. However, it is not yet clear whether these three categories are the same disease. The clinical presentations are diverse; the only common feature is the presence of pathologically proven Rosenthal fibers [5,6].

Owing to the discovery of inclusion bodies indistinguishable from Rosenthal fibers in fatal GFAP transgenic mice overexpressing human GFAP in astrocytes [7], de novo heterozygous mutations in the gene encoding GFAP, the main component of Rosenthal fibers, have been identified in patients with the infantile form of ALX [8]. *GFAP *gene mutations have also been identified in the juvenile [8,9] and adult [10,11] forms. These three clinically diverse forms are now widely accepted to be part of the same spectrum [12].

Each subtype has characteristic MRI findings. Cerebral white matter abnormalities, predominating in the frontal lobes, are typical of the infantile form of ALX [13], whereas nodular brainstem lesions and a kind of "garland" along the ventricular wall are seen, with contrast enhancement, in the juvenile form [14,15]. The adult form has a unique tadpole-like feature, caused by marked atrophy of the medulla oblongata and cervical spinal cord with an intact pontine base [11].

Here we present a case of sporadic adult-onset ALX with specific MRI findings: a typical tadpole-like brainstem, but also symmetric involvement of the basal ganglia, which is unusual in the adult form of ALX.

## Case Presentation

This Japanese patient, with an unremarkable past and no family history had been healthy until he was involved in a traffic accident at the age of 46. He suffered a bilateral brain contusion in the fronto-orbital areas, predominating on the left side. He did not lose his consciousness, but retrograde amnesia was seen. There was no hypoxia. After this accident, his character changed. He became querellous, could no longer manage his shop, and soon retired. He gradually became taciturn and his pronunciation became unclear, but he remained independent. At age 57, he began to drag his left foot as he walked, and this symptom gradually worsened. At the age of 59, progressive dysphagia appeared. He was referred to our hospital at the age of 60. Neurological examination revealed pseudobulbar palsy including aphonia, emotional incontinence and dysphagia, left-side predominant tetraparesis. The tone of the limb muscles was spastic, with bilateral positive Babinski signs. Axial rigidity, bradykinesia and retropulsion were observed, but no tremor or palatal myoclonus. Cerebellar ataxia was ambiguous because of spastic tetraparesis requiring use of a wheelchair, but bilateral gaze-evoked nystagmus was seen. He was clearly demented and angrily refused everything he was asked to do by shaking his head instead of speaking because of aphonia. Further evaluation of his dementia was impossible.

**Figure 1:**
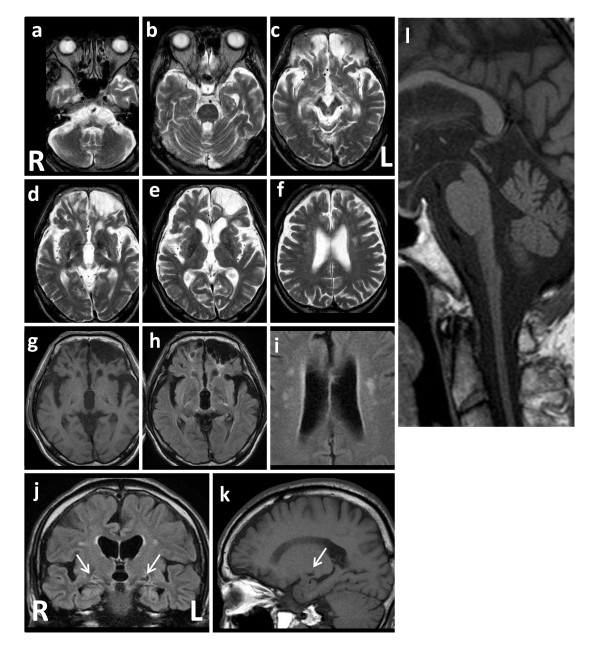
**Brain MRI of a patient with adult-onset Alexander disease**. a-f: T2-weighted axial images showing marked atrophy of the medulla oblongata (a) with slight cerebellar atrophy (a, b) but little atrophy of the pontine base (b), enlargement of the fourth ventricle (b), atrophy of the midbrain, especially the dorsal part (c), bilateral changes in the posterior part of globus pallidus (d), bilateral lesions of the fronto-orbital areas, predominating on the left, caused by brain contusion (d, e, g, h), moderate cortical atrophy with ventricular enlargement (e, f), and bilateral lacunae in the deep white matter, but no leukoencephalopathy (f). g and k: T1-weighted images of the lesions on axial (g) and sagittal (k) sections. h, i and j: FLAIR images of the lesions on axial (h, i) and coronal (j) sections. The lesions on coronal (j) and sagittal (k) sections are indicated by arrows. Note the absence of ventricular garlands [15]. l: T1-weighted sagittal section showing typical tadpole-like brainstem atrophy, consisting of marked cervico-medullary atrophy with an intact pontine base; note that atrophy of the midbrain tegmentum also contributes to the formation of the tadpole.

Laboratory tests, including hematology, routine blood chemistry, and analyses of urine and cerebrospinal fluid, were unremarkable. In addition to the bilateral contusion in the fronto-orbital areas, especially on the left side, brain MRI showed a tadpole-like brainstem, caused by marked atrophy from the medulla oblongata to the cervical spinal cord, sparing the pontine base. Marked atrophy of the midbrain tegmentum, mild cerebellar atrophy with a little enlargement of the fourth ventricle, and slight cerebral atrophy were also seen. The typical periventricular lesions (ventricular garlands [15]) and leukoencephalopathy were not seen, however, several lacunae were observed bilaterally in deep white matter. The posterior part of globus pallidus was involved bilaterally, as shown by a signal change without contrast enhancement (Figure 1).

With informed consent, the *GFAP *gene was sequenced, and a heterogeneous missense mutation was detected in exon 5 (c.827G>T), causing a change of arginine to leucine at amino acid position 276 (p.R276L). We have already described this mutation in a patient with pathologically proven hereditary adult-onset ALX [11]. There was no relationship between the present patient and the family previously reported [11]. According to an interview, however, both families originated from the same region of Japan.

## Discussion

We have described here a new patient with sporadic adult-onset ALX, and have identified a heterozygous missense mutation in the *GFAP *gene, c.827G>T, p.R276L, which was already shown to be pathogenic in a case of pathologically proven hereditary adult-onset ALX [11]. This mutation has not been seen for the last 7 years. Thus, this report reconfirms the pathogenetic nature of the mutation and the clinical picture of this form of the disease. So far, the phenotype associated with the R276L mutation is adult-onset spastic ataxia with pseudobulbar symptoms.

Following identification of the *GFAP *gene as responsible for ALX [8], the adult form has been increasingly reported [10,11,15-36]. However, only a few cases have been pathologically proven [11,16,17,21,28]; the rest were diagnosed as having ALX only by molecular testing. Since missense mutations may only be polymorphisms [37], their pathogenicity must be accepted with caution. Indeed, the E223Q mutation, identified in a patient with neurological deficits and radiological findings atypical for adult-onset ALX [38], is now classified as a polymorphism [21]. Therefore, to avoid this kind of confusion, it is worthwhile defining the typical presentation of adult-onset ALX, including age at onset, cardinal clinical symptoms, neuroradiological findings and clinical course, as described in published reports [see review article, ref [33]].

To date, at least 24 reports of adult onset ALX, including over 40 patients, with 22 different missense mutations in the *GFAP *gene, have been published (Table 1) [10,11,15-36]. There are no sex differences, and half of the cases are familial, consistent with autosomal dominant transmission. The mean age at onset is in the late thirties, although an asymptomatic carrier over age 60 has been described [30]. The cardinal triad of the clinical presentation is pseudobulbar or bulbar palsy, spastic paresis (usually hemiparesis, not paraparesis, at onset), and ataxia, each of which is observed in approximately 70% of patients. Palatal myoclonus is observed in only one third, although it is specific to ALX, and the key finding for a diagnosis, especially in hereditary cases [10]. Mental function is usually preserved, although our patient was obviously demented. Dementia cannot, however, be considered a symptom of adult-onset ALX in this patient, because of his cerebral contusion.

**Table 1:** Summary of the clinical features and MRI features of adult-onset Alexander disease reported in the literature [10,11,15-36].

Sex Difference	M/F = 23/22
Average age at onset	37.0 ± 17.9 (n = 36), Range: 12.5-62

Clinical features	

Bulbar symptom	35/45 (78%)

Pyramidal tract signs	33/45 (73%)

Ataxia	31/44 (71%)

Palatal myoclonus	15/38 (39%)

MRI findings	

Marked medullary atrophy	37/42 (88%)

Deep white matter abnormalities	21/43 (49%)

Brainstem signal change (including nodular lesions)	16/36 (44%)

As for the MRI findings [see review article, ref [34]], most of the cases had medullary abnormalities (either signal abnormalities or atrophy), and the marked tadpole-like atrophy of the medulla oblongata and cervical spinal cord with an intact pontine base [11]. We would like to emphasize that not only cervicomedullary atrophy with an intact pontine base, but also severe atrophy of the midbrain tegmentum contributes to the formation of the tadpole. This unusual atrophy is quite specific to adult-onset ALX, and 88% of the patients with adult-onset ALX in the literature showed marked medullary atrophy (Table 1). Thus, awareness of this MRI pattern allows effective selection of the patients who need genetic investigations for mutations in the *GFAP *gene [34]. Indeed, we could have diagnosed adult-onset ALX in the present patient on the basis of this form of brainstem atrophy.

Approximately half of the patients had deep white matter lesions or periventricular rims, although not always with frontal predominance as in infantile-onset ALX; the absence of these abnormalities is significantly associated with older age at onset (average age at onset; negative 43.7 ± 14.1 (n = 18) vs. positive 30.9 ± 12.8 (n = 18), *p *= 0.008), consistent with previous study [34]. Similarly, nodular lesions in the brainstem are observed in about half of the patients, and are significantly associated with a younger age at onset (average age at onset; positive 28.2 ± 11.8 (n = 13) vs. negative 43.6 ± 13.9 (n = 18), *p *= 0.003).

Besides the typical clinical and neuroradiological features, this case of adult-onset ALX is instructive because of the bilateral involvement of the basal ganglia. This is not uncommon in infantile or juvenile-onset ALX, and is one of the radiological criteria for the diagnosis [13], but has rarely been observed in the adult form of the disease. Symmetrical striatal lesions were observed in one patient, however, with hypointensity on T2-weighted MRI [22]. Basal ganglia lesions with hyperintensity on T2-weighted MRI, such as spotty lesions [19] and bilateral lesions in the lateral putamen [30], have occasionally been reported; both of these signs are ambiguous, however, and do not resemble those of our patient. Thus, the symmetrical lesions in our patient are interesting findings in adult-onset ALX, and might be related to a rigid-bradykinesia type parkinsonism, although this is uncertain because spastic paresis masks the parkinsonism. The clinical signs and symptoms of basal ganglia involvement, such as parkinsonism [patient 1 of ref [21]], diffuse bradykinesia [22], and rigidity of the arms [29] are rarely reported. The obvious parkinsonism reported in one patient was induced by valproate [32].

## Conclusion

We described here a new sporadic case of genetically confirmed adult-onset ALX. The tadpole-like brainstem atrophy is quite specific for adult-onset ALX. Thus, faced with a patient with progressive spastic ataxia, bulbar or pseudobulbar signs, and the typical tadpole on MRI, a genetic investigation of the *GFAP *gene is strongly recommended. The unusual feature of our patient is the obvious symmetrical involvement of the basal ganglia, which presumably caused a rigid-bradykinesia type parkinsonism. More cases must be studied to completely elucidate the characteristics of adult-onset ALX.

## Consent

Written informed consent was obtained from the patient and his wife for publication of this case report and accompanying images. A copy of the written consent is available for review by the Editor-in-Chief of this journal.

## Abbreviations

ALX: Alexander disease; GFAP: glial fibrillary acidic protein; FLAIR: fluid attenuated inversion recovery;

## Competing interests

The authors declare that they have no competing interests.

## Authors' contributions

This manuscript was drafted by MN. YT and IN contributed to the references and helped to write the manuscript. JH, HS and KS sequenced the *GFAP *gene. All authors contributed to the critical review and approval of the final draft.

## Pre-publication history

The pre-publication history for this paper can be accessed here:

http://www.biomedcentral.com/1471-2377/10/21/prepub
